# Relationships of Isolated Nocturnal Hypertension with Glomerular Filtration Rate and Albuminuria

**DOI:** 10.3390/diseases13040107

**Published:** 2025-04-02

**Authors:** Caterina Carollo, Giulio Geraci, Alessandra Sorce, Raffaella Morreale Bubella, Emanuele Cirafici, Maria Elena Ciuppa, Salvatore Evola, Giuseppe Mulè

**Affiliations:** 1 Unit of Nephrology and Dialysis, Hypertension Excellence Centre, Department of Health Promotion, Mother and Child Care, Internal Medicine and Medical Specialties (PROMISE), University of Palermo, 90133 Palermo, PA, Italy; caterina.carollo@unipa.it (C.C.); alessandra.sorce@community.unipa.it (A.S.); emanuele.cirafici@community.unipa.it (E.C.); giuseppe.mule@unipa.it (G.M.); 2 Department of Medicine and Surgery, “Kore” University of Enna, 94100 Enna, Italy; 3 Ophthalmology Operative Unit (UOC)–Specialized Ophthalmology Unit (UOS), ARNAS Civico Hospital, 90127 Palermo, PA, Italy; 4 Department of Health Promotion, Mother and Child Care, Internal Medicine and Medical Specialties, University of Palermo, 90133 Palermo, PA, Italy; mariaelena.ciuppa@community.unipa.it; 5 Catheterization Laboratory, Department of Medicine and Cardiology, Azienda Ospedaliera Universitaria Policlinico “P. Giaccone”, Via del Vespro 129, 90127 Palermo, PA, Italy; salvatore.evola@policlinico.pa.it

**Keywords:** hypertension, isolated nocturnal hypertension, INH, CKD, albuminuria, 24 h ABPM

## Abstract

Background/Objectives: Isolated nocturnal hypertension (INH) represents a unique phenotype that can only be identified through ambulatory blood pressure monitoring (ABPM). An increasing body of evidence suggests a significant association between INH and heightened cardiovascular morbidity, mortality, and, more recently, kidney disease progression. Considering these findings, this study aims to retrospectively assess the prevalence of INH and its relationship with glomerular filtration rate (GFR) and albumin excretion rate (AER) in a large cohort of hypertensive patients. Methods: A total of 1340 subjects selected from the patients of our European Hypertension Excellence Centre of the University of Palermo were enrolled. Biochemical tests, urinalysis, 24 h ambulatory blood pressure monitoring, and collection of anamnestic and anthropometric data were performed on each patient. Results: In our cohort, the prevalence of INH was 11%. Logistic regression analyses revealed that male sex, AER, and eGFR were significantly associated with the INH phenotype. AER ≥ 5.8 µg/min predicted the presence of INH with 73.7% sensitivity and 58.4% specificity. An eGFR < 60 mL/min/1.73 m^2^ was also correlated with INH, although its predictive value was less prominent. Multivariable regression models confirmed that AER and eGFR, along with male sex, were independent predictors of INH. In patients with normal blood pressure, AER and metabolic syndrome were also associated with INH. CKD (AER < 30 mg/day and eGFR < 60 mL/min/1.73 m^2^) was significantly linked to INH. Conclusions: Our research confirms the direct relationship between AER and INH and the inverse relationship between GFR and INH, thus underlining the leading role of renal function in the onset of INH, as widely observed in the literature. The confirmed association between renal markers and INH in the subgroup of subjects with a clinically normal blood pressure could help us to identify the subjects who should undergo ABPM.

## 1. Introduction

Isolated nocturnal hypertension (INH) was first described in 2007 by Li et al. as a distinct clinical entity characterized by elevated nocturnal blood pressure (BP) (>120 and/or 70 mmHg) despite normal daytime BP (<135/85 mmHg) [1]. INH represents a unique phenotype that can only be identified through ambulatory blood pressure monitoring (ABPM) and not by conventional clinical BP measurements. It is considered to be a prevalent subtype of masked hypertension, likely contributing to its overall occurrence.

Masked hypertension (MH) is not uncommon in the general population [2], and it is associated with increased organ damage, cardiovascular risk, and mortality [3]. When INH coexists with clinically normal BP values, it is termed masked nocturnal hypertension in untreated individuals or masked uncontrolled nocturnal hypertension in patients receiving antihypertensive treatment [1,4]. Notably, more than one-third of individuals with nocturnal hypertension are affected by INH [5,6].

The precise prevalence of INH remains incompletely understood due to variability in study populations and methodologies; however, it is estimated to be approximately 11%.

An increasing body of evidence suggests a significant association between INH and heightened cardiovascular morbidity and mortality [7,8]. In the International Database of Ambulatory Blood Pressure in relation to Cardiovascular Outcome (IDACO), among 8711 subjects examined, 577 were diagnosed with INH. These individuals exhibited a substantially greater risk of mortality and cardiovascular events compared to their normotensive counterparts [9].

Recent studies have reinforced the significant association between INH and increased cardiovascular morbidity and mortality. Building upon earlier findings, contemporary research continues to highlight the critical role of nocturnal blood pressure monitoring in identifying individuals at elevated risk.

A paper published in 2023 examined the impact of nocturnal hypertension on cardiovascular outcomes. The researchers found that individuals with elevated nighttime blood pressure had a higher incidence of adverse cardiovascular events and mortality compared to those with normal nocturnal blood pressure. This underscores the importance of monitoring and managing nocturnal blood pressure to mitigate cardiovascular risks [10].

In the context of chronic kidney disease (CKD), recent investigations have further elucidated the relationship between INH and adverse health outcomes. A study involving CKD patients demonstrated that nocturnal hypertension is prevalent in this population and is associated with an increased risk of cardiovascular events and progression of renal disease. In a cohort of 588 patients with chronic kidney disease (CKD), INH was linked to an elevated risk of both renal and cardiovascular events in comparison to individuals with normal nocturnal BP. This increased risk may be mediated by a higher prevalence of hypertension-mediated organ damage (HMOD) in affected individuals [11].

The findings suggest that nocturnal blood pressure patterns should be a key consideration in the management of CKD to prevent further organ damage.

A 2021 study investigated the relationship between circadian blood pressure (BP) variations and arterial stiffness in patients with chronic kidney disease (CKD) [12]. The findings demonstrated that altered circadian BP rhythms, particularly a blunted nocturnal BP decline, were associated with increased arterial stiffness, a well-established predictor of cardiovascular events. These results underscore the necessity of evaluating and optimizing nocturnal BP management to mitigate cardiovascular risk in CKD patients.

Similarly, in 2022, Borrelli et al. examined the impact of nocturnal BP patterns on CKD progression, revealing that patients exhibiting a non-dipping nocturnal BP pattern had a significantly greater risk of disease progression compared to those with a preserved dipping pattern. These findings highlight the critical need for targeted therapeutic interventions to regulate nocturnal BP and decelerate renal function decline [13].

Collectively, these studies emphasize the pivotal role of nocturnal BP monitoring in CKD management. The implementation of ambulatory BP monitoring for nocturnal BP assessment can facilitate the early identification of high-risk patients and enable the development of individualized treatment strategies to optimize both cardiovascular and renal outcomes.

Considering these findings, the present study aims to retrospectively assess the prevalence of INH and its relationship with glomerular filtration rate (GFR) and albumin excretion RATE (AER) in a large cohort of individuals referred to our Regional Referral Center for Arterial Hypertension.

## 2. Materials and Methods

We enrolled 1340 subjects, selected from the patients of our European Hypertension Excellence Centre of the University of Palermo. The enrolled patients had a suitable and valid ABPM prolonged for 24 h.

ABPM was performed during a working day. Endocrine reasons for hypertension were ruled out by means of biochemical and instrumental evaluations.

The exclusion criteria were as follows: defective or excessive urine collection; endocrine or malignant hypertension; ABPM showing less than 80% effective measurements and missing recordings for more than 1 h; acute kidney injury; heart failure; personal history or clinical signs of ischemic heart disease and cerebrovascular disease; major non-cardiovascular diseases.

Office BP was considered as the mean of three consecutively measurements obtained at 2 min intervals by an electronic oscillometric validated device (WatchBP Office, Microlife Corporation, Taipei, Taiwan) after 5 min of rest in a sitting position.

Routine biochemical parameters were measured with an autoanalyzer by standard techniques. Creatinine levels were obtained by a standardized enzymatic evaluation.

In patients with urinalysis showing proteinuria, even in trace amounts, or microalbuminuria detected via semi-quantitative dipstick evaluation, a 24 h urinary albumin excretion assay was requested. Albuminuria was measured using a turbidimetric method and expressed in mg/day. Serum creatinine levels were determined using a standardized enzymatic method (Creatinine Plus, Roche Diagnostics). The glomerular filtration rate (GFR) was estimated using the CKD-EPI (Chronic Kidney Disease Epidemiology Collaboration) equation.

Patients with CKD were classified into five stages according to the 2012 KDIGO guidelines on chronic kidney disease. Due to the small number of subjects in stage V, we grouped patients of stages IV and V [14].

Twenty-four-hour urine was collected in a resting, non-working day: patients were advised to avoid physical stresses. The exam was repeated in case of fever or urinary tract infections. We defined patients with a 24 h AER > 20 mcgmin (that is, 30 mg/die) as “albuminuric” [14].

### 2.1. Twenty-Four-Hour Ambulatory Blood Pressure Monitoring

To obtain a 24 h ABPM, we used a non-invasive, portable recording device (SpaceLabs 90207 recorder, Redmond, WA, USA). Blood pressure was measured every 15 min during the day (from 7:00 to 22:00) and every 20 min overnight (from 22:00 to 7:00). To read and analyze the data, we used the interface software SpaceLabs ABP90209, version 2.40.23. Recordings showing a systolic blood pressure > 260 mmHg or <70 mmHg and a diastolic blood pressure > 150 mmHg or <20 mmHg were automatically excluded.

### 2.2. Statistical Analysis

Statistical analysis was performed with IBM SPSS version 26 software (IMB Corp, Armonk, NY, USA)

The Kolmogorov–Smirnov test confirmed a normal distribution for all of the examined variables, with the exception of AER, hypertension duration, and blood triglyceride levels, which showed a positively asymmetrical distribution, so we modified these values as medians, interquartile ranges, and logarithms before starting any further analysis.

All of the continuous, normally distributed variables were expressed as means ± SD.

Differences in continuous variables among the groups were evaluated by means of Analysis of Variance (ANOVA) and, if the F test was significant, comparisons with normotensive subjects were obtained with the post hoc Dunnett test. The X2 test with Yates correction was applied to test the differences between the groups for categorical variables.

We used simple and multiple logistic regression to test the associations between INH (dependent variable) and other parameters. Regression was performed by creating some models in which AER, in addition to other confounding factors, was considered as a dichotomous variable (AER < 20 or >20 mcgmin, identified as 0 or 1, respectively) in model 1, whilst in model 2 it was considered as a continuous variable (such as an increase in its standard deviation). Finally, in model 3, to make AER a dichotomous variable, we employed a cut-off value derived from ROC curve analysis of the data to identify the threshold AER level that could better detect INH.

ROC curves derived from every model of multiple regression were further compared by means of Z tests.

The null hypothesis was rejected in every two-tailed test, with a *p* < 0.05.

## 3. Results

The 1340 enrolled patients were divided into four phenotypes on the basis of diurnal and nocturnal ABPM recordings: daily and nocturnal normotensive (DNN) subjects, patients with isolated diurnal hypertension (IDH) or isolated nocturnal hypertension (INH), and, finally, people affected by daily and night hypertension (DNH). The prevalence of INH was 11%. In the groups with INH and DNH, male patients were more frequent than in the DNN group. Age, body mass index (BMI), waist circumference, blood glucose and lipid levels, and diabetes prevalence did not significantly differ among the four phenotypes.

The presence of a BMI > 25 kg/m^2^ did not differ among the four groups, even though obese subjects were more present (39%) in the INH group than in the DNN group (Table 1).

We observed that hypertension duration, the percentage of patients who took blood-pressure-lowering drugs, clinical and ABPM 24 h systolic and diastolic blood pressure, and 24 h heart rate were increased in the group of patients with elevated blood pressure compared with DNN subjects (see Table 2). The same table shows an increase in clinical diastolic blood pressure and average systolic and diastolic blood pressure in INH patients in comparison with DNN subjects.

**Figure 1:**
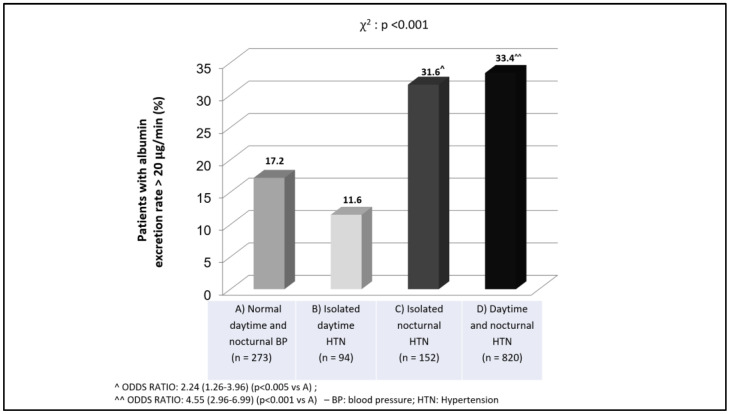
Distribution of albumin excretion rate among the different ABPM phenotypes of the study population.

By subdividing the entire population on the basis of the presence of CKD, as diagnosed by means of the KDIGO criteria, we observed that CKD patients, independent of their stage, were more present in the DNH (33.4%) and INH (31.6%) groups when compared with DNN (17.2%) and IDN (11.5%) (Figure 1).

We also found an increased chance to find CKD patients in the INH (odds ratio 2.22, *p* = 0.007) and DNH (OR 2.99 *p* > 0.001) groups than in the DNH group.

CKD stages 1 and 2 were significantly more present in the DNH group than in the DNN group (Table 3).

As described in Table 4, the median AER, the percentage of subjects with AER > 20 mcgmin, the amount of patients with GRF > 60 mL/min/1.73 mq (also expressed as percentage), and the mean creatinine levels were increased (with consequent lower GFR levels) in the INH group in comparison with DNN subjects.

The same parameters showed a similar trend in the comparison between the DNH and DNN groups, with the exception of a non-significant difference in creatinine levels.

We found the same trend in median AER, prevalence of pathological AER, and mean GFR values between the INH and DNN groups when we analyzed the subgroup with normal systolic and diastolic blood pressure, which represented 20% (N = 273) of the entire population (Table 5).

**Figure 2:**
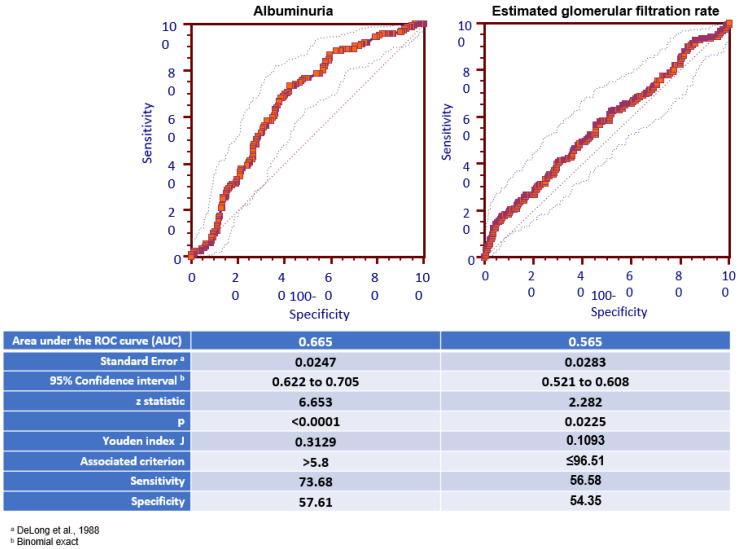
Receiver operating characteristic (ROC) curve of albuminuria and estimated glomerular filtration rate for predicting INH [15].

In a successive step, after excluding DNH subjects, we tested the ability of AER and eGFR to predict the presence of the INH phenotype by means of the C statistical test and ROC curves. AER showed an AUC value of 0.665 (*p* > 0.001), and 5.8 mcg min was the best cut-off to predict the presence of INH, with a sensitivity of 73.68% and a specificity of 57.61% (Figure 2). On the contrary, eGFR was not able to predict the presence of INH.

After logistical univariate regression, male sex, AER, and eGFR (considered as continuous variables, as increased standard deviations of the same variables, or as dichotomous variables) were significantly associated with INH. In fact, we found a threshold eGFR value of 60 mL/min/1.73 m^2^ and a threshold AER value of 20 mcgg/min.

By considering 5.8 microg/min as a cut-off value, AER was associated with INH. Antihypertensive drugs and clinical diastolic blood pressure also showed a positive relationship with INH; however, this association did not reach statistical significance.

**Figure 3:**
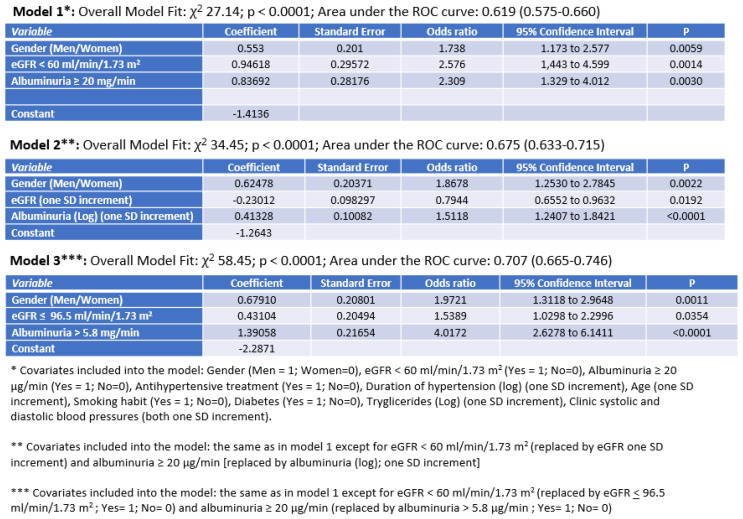
Independent correlates of isolated nocturnal hypertension in multiple logistic regression analyses.

In our models of multiple logistic regression, AER and eGFR were confirmed to be associated with INH, like the male sex. It is worthy of attention that model 3, in addition to confirming the results obtained with the other two models, showed the greatest predictive ability (Figure 3).

In the subgroup of subjects with normal office blood pressure, AER and metabolic syndrome were found to be significantly associated with INH.

The presence of CKD (as defined as AER < 30 mg/die and eGFR < 60 mL/min/1.73 m^2^) was significantly associated with INH (OR 2.57, 95% CI 1.443–4.599, *p* = 0.0014).

## 4. Discussion and Conclusions

In this study, the prevalence of INH was 11%. Among hypertensive subjects, INH was more frequent in males and in subjects with increased AER and reduced GFR.

Our observed prevalence is consistent with the results of Salazar et al., who found an INH prevalence of 12.9% in 1344 Argentinian subjects, and it was lower in patients classified as hypertensive after an office BP measurement than in normotensive subjects (7.4% vs. 17.2%; *p* < 0.001) [5].

In a retrospective analysis of the IDACO study population, INH was more prevalent in Black South African (10.5%) and Japanese subjects (10.2%) than in Western (6%) and Eastern European persons (7.9%).

Recently, Rhee found a prevalence of 22.8% in the general population [6].

These different prevalences could be due to the different ethnic groups, personal details, and non-homogeneous structural and demographic characteristics of the populations included in the studies. Selection bias could also justify these results.

An INH prevalence of 11.4% (very similar to our findings) is described in the PAMELA study that examined 2020 subjects in Italy [16].

In the Jackson Heart Study, the prevalence of INH was 19% in African-American subjects. This datum, higher than the other mentioned findings, could be due both to the increased number of non-dipping subjects and to a greater susceptibility of these subjects to renal damage, even though it was clinically silent. In the same study, a relationship between urinary protein excretion and INH was found, but it was not statistically significant at the multivariate analysis after correction for confounding factors. This observation could be due to the small sample size (425 patients) [17].

Other studies have underlined the role of the association between precocious alterations in renal function, nocturnal increases in arterial blood pressure, and a dysregulation of circadian blood pressure trends [18,19,20]. In 29 teenagers who underwent renal transplantation, McGlothan KR et al. [21] observed that INH was more prevalent than daily hypertension: isolated diastolic nocturnal hypertension was present in 51% of the cases, while IDDH was observed in 10% of subjects. Similar results were observed by Pais [22] in pediatric renal transplant recipients in Southern India. In these patients, the increased nocturnal arterial BP could be due not only to kidney disease-related mechanisms, likely prolonged after transplantation, but also to steroid and immunosuppressive agents employed to prevent rejection.

In 2015, Wang et al. [23] found an INH prevalence of 20.4% in 1282 hospitalized CKD patients, similar to what was observed in the Jackson Heart Study. In this population, INH was independently associated with age, GFR, and clinical diastolic BP. Such an increased prevalence could be due to the peculiar population, exclusively represented by CKD patients.

A relationship between kidney disease and INH has already been documented in childhood, as suggested in 198 Japanese children and teenagers. In this population, INH was found in 32 subjects (16%) [24].

A similar datum was observed in the multicentric Cardiovascular Comorbidity in Children with Chronic Kidney Disease Study [25], which enrolled 456 European child and teenage renal patients (stage III and IV). In this population, the prevalence of INH was 13.4%. Age, height, small birth weight, PTH levels, and a shorter duration of kidney disease were associated with INH [26].

Our research confirms the direct relationship between AER and INH and the inverse relationship between GFR and INH, thus underlining the leading role of renal function in the onset of INH, as widely observed in the literature. As a matter of fact, renal function and nocturnal blood pressure are largely recognized to be associated, but this phenomenon is not yet deeply understood. Among the different mechanisms that could contribute to this relationship, we have to mention an impaired daily natriuresis that makes nocturnal blood pressure elevated to compensate for diminished natriuresis by pressure natriuresis [18]. Moreover, increased sympathetic activity, already well proven in different kidney diseases long before a clinical condition, could play an important role [27,28,29]. Other important mechanisms could include altered baroreflex sensitivity [30], an autonomic dysfunction, leading to clinostatic hypertension and postural hypotension [31]. Another crucial factor is the presence or the absence of sleep apnea syndrome, which is more frequent in ESKD [32].

Hypertension-lowering drugs with a reduced half-life are worthy of mentioning: if taken in the early morning, they do not guarantee 24 h effectiveness [33].

Our investigation, being a retrospective analysis, did not allow us to completely explain the reasons for the associations that we describe here.

The lack of data about sleep apnea syndrome (such as polysomnography, Berlin questionnaire, or Epworth score) and the lack of ethnic heterogeneity are the main limits of this study.

Another interesting finding is the INH prevalence (17%) observed in the subgroup of patients with a clinically normal blood pressure. This prevalence, greater than what was observed in the entire population, underlines the fundamental role of masked hypertension.

The confirmed association between renal markers and INH in the subgroup of subjects with a clinically normal blood pressure could help us to identify the subjects who should undergo ABPM. Such a preventive strategy could allow us to precociously identify and treat a serious clinical condition and its related cardiorenal risks.

## Figures and Tables

**Table 1:** Main clinical characteristics of the study population divided according to different ABPM phenotypes.

	Ambulatory Blood Pressure Phenotypes				
	(A) Normal Daytime and Nocturnal BP (n = 273)	(B) Isolated Daytime HTN (n = 94)	(C) Isolated Nocturnal HTN (n = 152)	(D) Daytime and Nocturnal HTN (n = 820)	ANOVA or X2 Test (p)
Gender (Men) (%)	45.1	56.8	61.2 ^	62.6 ^^	<0.0001
Age (years)	46.4 ± 14.1	46 ± 13.3	47.9 ± 14.9	47.1 ±11.9	0.59
Smokers (%)	23	31.5	23.5	31.2 *	0.045
Body mass index (Kg/m^2^)	28.6 ± 5.3	28.8 ± 4.4	28.3 ± 4.8	28 ± 4.1	0.64
Waist circumference (cm)	97.3 ± 12.4	100.2 ± 10	94.7 ± 14.6	96.2 ± 12.1	0.83
Total cholesterol (mg/dL)	206.4 ± 41.3	204.2 ± 38.1	211.3 ± 44.7	206.1 ± 41.1	0.56
HDL cholesterol (mg/dL)	47.4 ± 11.1	46.5 ± 9.1	44.7 ± 11.6	45.5 ± 10.2	0.11
Triglycerides (mg/dL)	125 (84–170)	108 (84–162)	125 (81–169)	127 (89–182)	0.06
LDL cholesterol (mg/dL)	131.8 ± 39.1	130.9 ± 35.9	135.9 ± 42.2	134.4 ± 37.8	0.7
Serum glucose (mg/dL)	95.8 ± 22.1	95.2 ± 18.2	95.6 ± 20.1	96.2 ± 19.8	0.83
Diabetes (%)	6.3	9	9	6.7	0.68
Metabolic syndrome (%)	34.8	38.9	47.4	51.7	<0.0001

* *p* < 0.05; ^ *p* < 0.005; ^^ *p* < 0.001 vs (A); BP: blood pressure; HTN: hypertension.

**Table 2:** BP parameters and heart rates of the study population divided according to different ABPM phenotypes.

	Ambulatory Blood Pressure Phenotypes				
	(A) Normal Daytime and Nocturnal BP (n = 273)	(B) Isolated Daytime HTN (n = 94)	(C) Isolated Nocturnal HTN (n = 152)	(D) Daytime and Nocturnal HTN (n = 820)	ANOVA or X^2^ Test (*p*)
Duration of hypertension (months)	12 (7–24)	12 (6–36)	12 (8–24)	24 (11–48) *	<0.001
Drug therapy for hypertension (%)	70.2	75.8	79.3	83.2 ^^	<0.001
Clinic systolic BP (mmHg)	141 ± 17.5	151 ± 18.1 ^^	144 ± 16.6	156 ± 20.5 ^^	<0.001
Clinic diastolic BP (mmHg)	84 ± 12.3	91 ± 11.9 ^^	88 ± 13.5 *	95 ± 15.8 ^^	<0.001
24 h systolic BP (mmHg)	118 ± 6.4	127 ± 6.8 ^^	125 ± 5.7 ^^	138 ± 11.6 ^^	<0.001
24 h diastolic BP (mmHg)	84 ± 12.3	91 ± 11.9 ^^	88 ± 13.5 ^^	95 ± 15.8 ^^	<0.001
24 h heart rates (b/min)	73 ± 9.1	76 ± 7.6 *	73 ± 9.4	75 ± 10.3 ^^	0.001
Daytime systolic BP (mmHg)	122 ± 6.9	135 ± 7.9 ^^	126 ± 5.6 ^^	144 ± 10.7 ^^	<0.001
Daytime diastolic BP (mmHg)	75 ± 6.4	86 ± 6.7 ^^	79 ± 5.0 ^^	95 ± 15.8 ^^	<0.001
Nighttime systolic BP (mmHg)	109 ± 7.9	110 ± 6.4	121 ± 8.5 ^^	131 ± 12.8 ^^	<0.001
Nighttime diastolic BP (mmHg)	62 ± 5.6	65 ± 5.3 **	74 ± 5.2 ^^	81 ± 9.5 ^^	<0.001

* *p* < 0.05; ** *p* < 0.01; ^^ *p* < 0.001 vs (A); BP: blood pressure; HTN: hypertension.

**Table 3:** Distribution of CKD stages among the different ABPM phenotypes of the study population.

	Ambulatory Blood Pressure Phenotypes				
	(A) Normal Daytime and Nocturnal BP (n = 273)	(B) Isolated Daytime HTN (n = 94)	(C) Isolated Nocturnal HTN (n = 152)	(D) Daytime and Nocturnal HTN (n = 820)	ANOVA or X^2^ Test (*p*)
No CKD (%) (n = 919)	82.8	88.4	68.4 ^	61.6 ^^	<0.0001
Stage 1 CKD (%) (n = 187)	5.5	3.2	9.2	18.9 ^^	<0.0001
Stage 2 CKD (%) (n = 89)	3.7	2.1	4.6	8.5 **	0.005
Stage 3a CKD (%) (n = 66)	3.7	4.2	6.6	5.1	0.5749
Stage 3b CKD (%) (n = 45)	2.2	1.1	5.9	3.5	0.1194
Stage 4-5 CKD (%) (n = 34)	2.2	1.1	5.3	2.3	0.127

NO CKD: ^ ODDS RATIO: 2.22 (1.40–3.5) (*p* = 0.007 vs A) ; ^^ ODDS RATIO: 2.99 (2.26–4.23) (*p* < 0.001 vs A); Stage 1 CKD: ODDS RATIO: 0.25 (0.14–0.43) (*p* < 0.001 vs A); Stage 2 CKD: ** ODDS RATIO: 0.41 (0.21–0.80) (*p* = 0.007 vs A); BP: blood pressure; HTN: Hypertension.

**Table 4:** Renal parameters of the study population divided according to different ABPM phenotypes.

	Ambulatory Blood Pressure Phenotypes				
	(A) Normal Daytime and Nocturnal BP (n = 273)	(B) Isolated Daytime HTN (n = 94)	(C) Isolated Nocturnal HTN (n = 152)	(D) Daytime and Nocturnal HTN (n = 820)	ANOVA or X^2^ test (*p*)
Albuminuria (μg/min)	5.0 (3.0–9.7)	5.6 (3.3–10.0)	9.1(5.4–17.9) ^^	14.7(8–30.3) ^^	<0.0001
Patients with high albuminuria (%)	9.5	7.4	19.1 ^	32.3 ^^	<0.0001
Serum creatinine (mg/dL)	0.89 ± 0.42	0.87 ± 0.29 *	1.06 ± 0.67 ^	0.99 ± 0.52	0.002
Estimated GFR (ml/min/1.73 m^2^)	93.6 ± 24.2	95.8 ± 20.8 ^	87.4 ± 28.8 *	89.7 ± 23.4 *	0.005
Patients with eGFR 60 ml/min (%)	8.1	6.3	17.8 ^	11	0.01

* *p* <0.05; ^ *p* <0.005; ^^ *p* < 0.001 vs (A).

**Table 5:** Renal parameters of a subgroup of the study population with normal office BP, divided according to different ABPM phenotypes.

	Ambulatory Blood Pressure Phenotypes				
	(A) Normal Daytime and Nocturnal BP (n = 102)	(B) Isolated Daytime HTN (n = 8)	(C) Isolated Nocturnal HTN (n = 47)	(D) Daytime and Nocturnal HTN (n = 106)	ANOVA or X^2^ Test (*p*)
Albuminuria (μg/min)	5.0 (3.0–11)	3.4 (2.0-3.9)	9.7 (6.0–19.0) *	10.5 (7.4–20.7) ^^	<0.0001
Patients with high albuminuria (%)	8.8	0	21.3 *	24.5 ^	0.002
Serum creatinine (mg/dL)	0.91 ± 0.55	0.88 ± 0.37 *	1.23 ± 0.97 ^	1.05 ± 0.43	0.02
Estimated GFR (mL/min/1.73 m^2^)	97.4 ± 26.4	95.0 ± 23.5 ^	82.8 ± 33.4 ^	86.1 ± 24.9 ^	0.004
Patients with eGFR 60 mL/min (%)	9.8	12.5	21.3	15.1	0.194

* *p* < 0.05; ^ *p* < 0.005; ^^ *p* < 0.001 vs (A).

## Data Availability

Data are available upon reasonable request.
